# Detection of consensus genomic regions and candidate genes for quality traits in barley using QTL meta-analysis

**DOI:** 10.3389/fpls.2023.1319889

**Published:** 2024-01-11

**Authors:** Binbin Du, Jindong Wu, Meng Wang, Jia Wu, Chaoyue Sun, Xingen Zhang, Xifeng Ren, Qifei Wang

**Affiliations:** ^1^College of Biotechnology and Pharmaceutical Engineering, West Anhui University, Lu’an, Anhui, China; ^2^Xingtai Agriculture and Rural Bureau, Xingtai, Hebei, China; ^3^Hubei Hongshan Laboratory, College of Plant Science and Technology, Huazhong Agricultural University, Wuhan, Hubei, China; ^4^Institute of Crop and Nuclear Technology Utilization, Zhejiang Academy of Agricultural Sciences, Hangzhou, Zhejiang, China

**Keywords:** barley, quality traits, QTL meta-analysis, MQTL, candidate gene

## Abstract

Improving barley grain quality is a major goal in barley breeding. In this study, a total of 35 papers focusing on quantitative trait loci (QTLs) mapping for barley quality traits published since 2000 were collected. Among the 454 QTLs identified in these studies, 349 of them were mapped onto high-density consensus maps, which were used for QTL meta-analysis. Through QTL meta-analysis, the initial QTLs were integrated into 41 meta-QTLs (MQTLs) with an average confidence interval (CI) of 1. 66 cM, which is 88.9% narrower than that of the initial QTLs. Among the 41 identified MQTLs, 25 were subsequently validated in publications using genome-wide association study (GWAS). From these 25 validated MQTLs, ten breeder’s MQTLs were selected. Synteny analysis comparing barley and wheat MQTLs revealed orthologous relationships between eight breeder’s MQTLs and 45 wheat MQTLs. Additionally, 17 barley homologs associated with rice quality traits were identified within the regions of the breeder’s MQTLs through comparative analysis. The findings of this study provide valuable insights for molecular marker-assisted breeding and the identification of candidate genes related to quality traits in barley.

## Introduction

With the global population growing and living standard improving, the demand for food is constantly increasing. Barley, as an important cereal crop, is widely cultivated worldwide (Baik and Ullrich, 2008). Apart from being consumed directly as a ration, barley is also ultilized for brewing beer and making various food products such as pasta, pastries, and cookies (Kochevenko et al., 2018). Alongside efforts to increase barley yield, the improvement of quality traits has gained increasing attention. Nevertheless, enhancing quality traits in barley remains challenging due to the fact that they are controlled by multiple genes and are susceptible to environmental factors (Hayter and Riggs, 1973; Coles et al., 1991; Fox et al., 2003).

Barley quality is characterized by a range of crucial traits, including protein content, amylose content, starch pasting characteristics, and malt quality traits such as malt extract, wort viscosity, kolbach index, free α-amino nitrogen, and diastatic power (Burger and Laberge, 1985; Brennan et al., 1996; Baik and Ullrich, 2008). Understanding the genetic basis of these traits is pivotal for enhancing barley varieties. A comprehensive knowledge of the genetic mechanisms underlying these traits is indispensable for breeding high-quality barley varieties. To date, numerous researchers have conducted QTL mapping studies on barley quality traits (Marquez-Cedillo et al., 2000; Gao et al., 2004; Von Korff et al., 2008; Wang et al., 2015; Kochevenko et al., 2018; Huang et al., 2021). However, differences in mapping populations, molecular markers, and experimental environments across these studies contribute to variations in QTL results (Zhang et al., 2017). Therefore, accurately pinpointing QTL locations and identifying candidate genes for barley quality traits remains challenging.

Meta-analysis is a powerful tool to synthesize the findings of multiple independent studies (Egger et al., 1997). QTL meta-analysis integrates initial QTLs from diverse studies into a consensus map, which helps to narrow down the confidence intervals of MQTLs and enhance the detection accuracy and prediction precision of MQTLs (Goffinet and Gerber, 2000). Nowadays, QTL meta-analysis has been widely utilized to study salt tolerance and yield -related traits in rice (Khahani et al., 2020; Mansuri et al., 2020), abiotic stress tolerance, yield, quality, and flag leaf morphology in wheat (Du et al., 2022; Gudi et al., 2022; Saini et al., 2022; Tanin et al., 2022), yield and popping traits in maize (Wang et al., 2020; Kumar et al., 2021), and agronomic traits, disease resistance, and seed quality traits in pigeonpea (Halladakeri et al., 2023).

Until now, no QTL meta-analysis studies on barley quality traits have been reported. The objective of this study was to use QTL meta-analysis to integrate and comprehensively analyze all the QTL for barley quality traits published since 2000, and to validate the precision of the MQTL using GWAS results to identify key genomic regions and candidate genes that influence barley quality traits. The results of this study will enhance our understanding of the genetic mechanism underlying barley quality traits, providing an important foundation for improving barley quality and facilitating molecular marker-assisted selection.

## Materials and methods

### Data collection for QTL meta-analysis

A comprehensive collection and screening of QTL studies related to quality traits in barley from 2000 to the present identified 35 publications that provided the initial QTL information suitable for QTL meta-analysis. The basic information on population parents, type, size, traits involved, and molecular marker types for each study were listed in **Supplementary Table S1**. Each initial QTL was collected for related traits, flanking or closely linked markers, confidence intervals (CI, 95%), LOD values, and phenotypic variance explained (PVE) or R^2^ values (**Supplementary Table S2**). For initial QTL with missing LOD values in individual studies, a LOD value of 3 was assumed, and initial QTL with missing R^2^ values was ignored. Additionally, depending on the mapping population, the CI of the initial QTL was required to be recalculated according to the following equations: (1) double-haploid (DH) population, CI = 287/(population size × PVE); (2) recombinant inbred line (RIL) population, CI = 163/(population size × PVE); (3) F_2_ and backcross (BC) population, CI = 530/(population size × PVE) (Darvasi and Soller, 1997; Guo et al., 2006). The initial QTL mainly affected barley quality traits, including malt quality traits, flour pasting properties, and other quality traits. The detailed quality trait types and abbreviations were listed in **Supplementary Table S3**.

### Construction of consensus map

Construction of a consensus map was carried out by integrating and assembling reference genetic maps using the R package LPmerge (Endelman and Plomion, 2014). Six genetic linkage maps, namely “Barley, Consensus 2006, Marcel” (Marcel et al., 2006), “Barley, Consensus 2006, DArT” (Wenzl et al., 2006), “Barley, Consensus 2007, SSR” (Varshney et al., 2007) and “Barley, OPA 2009, Consensus” (Close et al., 2009) downloaded from the GrainGenes website (http://wheat.pw.usda.gov), along with InDel markers integrated with SSR, DArT and SNP markers for barley genetic map (Zhou et al., 2015) and “Barley 50k iSelect SNP Array” (Bayer et al., 2017), were utilized. The detailed scripts are provided in **Supplementary Data 1**. Furthermore, ten genetic maps were selected from 35 studies and integrated into the reference map using the iterative maps compilation tool of BioMercator v4.2.2 software to construct the final consensus map (Sosnowski et al., 2012) (**Supplementary Table S4**).

### QTL projection and QTL meta-analysis

Initial QTL was projected onto the consensus map using the QTLProj tool of the BioMercator V4.2 software. The input file formats for the initial QTL and the consensus map were listed in **Supplementary Tables S5**, **S6**. Then, QTL meta-analysis was performed on individual chromosomes using the two-step meta-analysis method of Veyrieras et al. (Veyrieras et al., 2007). The first step, QTLClust, clusters the initial QTL using five models: Akaike information criterion (AIC), AIC correction (AICc), AIC 3 candidate models (AIC3), Bayesian information criterion (BIC) and average weight of evidence (AWE), with the most frequent value calculated by the five models considered to be the optimal number of meta-QTLs on each chromosome. The second step, MQTLView, based on the number of MQTL determined in the last step, the peak and confidence interval of each MQTL on the consensus map were calculated. The LOD scores and PVE values of each MQTL were calculated from the corresponding mean values of all the initial QTLs it contained. BLASTN of the flanking marker sequences of the MQTL with the barley reference genome sequence (MorexV3) (Mascher et al., 2021) to obtain the physical location of the MQTL. Primer sequence information for AFLP, RFLP and SSR markers were obtained from grain genes (https://wheat.pw.usda.gov/GG3), sequence information for DArT markers was obtained from https://www.diversityarrays.com, SNP marker sequences were obtained from the studies of Close et al. (Close et al., 2009) and Bayer et al. (Bayer et al., 2017). The flanking markers of the MQTL were mapped to the barley MorexV3 genome by Barleymap (https://barleymap.eead.csic.es) (Cantalapiedra et al., 2015).

### Validation of MQTL with MTAs identified in GWAS

To verify the accuracy of the MQTL regions in this study, we collected important marker-trait associations (MTAs) information from nine GWAS studies on quality-related traits in barley published from 2015 to the present Details of the relevant traits, population type and size, and number of MTAs involved in these GWAS studies were listed in **Table 1**. We obtained the physical location of these MTAs in GWAS by comparing their sequence information with the barley MorexV3 genome using Barleymap (Cantalapiedra et al., 2015). MQTLs that co-located with at least one MTA were considered GWAS-validated MQTLs based on the comparison of their physical locations with those of the MTAs.

**Table 1:** GWAS information on barley quality traits for validation of MQTLs.

No	Source of genotype	Population size	Trait^a^	Marker type/number	Number of MTA^b^	Environment	Reference
1	spring barley breeding lines	4976	ME, GPC, WP, ST, DP, AA, BG	SNP/3072	215	USDA at Aberdeen, University of Minnesota, North Dakota State University, Oregon State University, Montana State University, Washington State University	(Mohammadi et al., 2015)
2	landraces, ICARDA lines, Ethiopian lines, NDSU lines, Kenyan cultivars	236	AA, DP, ME, FAN, KI, SP, BG, VIS	SNP/54515	106	Bekoji, Ethiopia	(Daba et al., 2018)
3	cultivated barley accessions	343	AA	SNP/1536	22	Okayama University, Kurashiki, Japan	(Sato et al., 2018)
4	spring and winter barley	407, 352	FRI, DP, AA, ME, PSY, FERM, MTN, SNC, VIS, BG,	SNP/22748; SNP/25575	15	UK	(Looseley et al., 2020)
5	International Barley Core Selected Collection	100	BG	SNP/279515	14	Changxing, China; Cixi, China	(Geng et al., 2021)
6	Two-rowed and six-rowed spring barley cultivars	660	BG, GPC, GSC, EX	SNP/2344	20	Kazakh Research Institute of Rice-growing, Kyzylorda, Kazakhstan	(Genievskaya et al., 2021)
7	Two-rowed and six-rowed spring barley cultivars	658	GPC, GSC, EX	SNP/1920	41	Karabalyk; Karaganda; Kyzylorda, Kazakhstan	(Genievskaya et al., 2022)
8	two-rowed spring barley accessions	406	GCC, GLC, GPC, GSC	SNP/1648	71	Kazakh Research Institute of Agriculture and Plant Growing, Kazakhstan	(Genievskaya et al., 2023)
9	NSGC Barley Core Panel	169	BG, DP, GPC, SP, ST, ME	SNP/5716	61	Bozeman Post Farm, State of Montana, USA	(Jensen et al., 2023)

a AA, α-amylase activity; BG, β-glucan; DP, diastatic power; EX, grain extractivity; FAN, free amino nitrogen; FERM, fermentability; FRI, friability; GCC, grain cellulose content; GLC, grain lipid content; GPC, grain protein concentration; GSC, grain starch content; KI, kolbach Index; ME, malt extract; MTN, malt total nitrogen content; PSY, predicted spirit yield; SNC, soluble nitrogen content; SP, soluble protein; ST, soluble/total protein; VIS, viscosity; WP, wort protein.

b Marker-trait association number (MTA) detected in previous GWAS studies.

### Orthologous MQTL analysis

To access the most stable MQTLs for barley quality traits, we conducted an analysis using MQTLs previously detected for quality traits in wheat (Gudi et al., 2022) to explore orthologous regions for barley and wheat quality traits. The analysis followed these steps: (1) gene models detected within the breeder’s MQTL region with physical intervals of less than 20 Mb were BLASTed against the wheat reference genome to identify wheat orthologs; (2) the physical locations of wheat orthologs were compared to wheat MQTL regions (Gudi et al., 2022); and (3) wheat MQTLs with at least four corresponding genes were considered as orthologous MQTL (OrMQTL).

### Homology-based candidate gene identification within breeder’s MQTLs region

To identify candidate gene (CGs) within the breeder’s MQTLs, specific criteria proposed by Löffler et al. (2009) were applied. Breeder’s MQTLs were selected based on the following criteria: genetic distance < 2 cM, initial QTL number of at least four from different studies, and MQTLs with PVE > 10%. The Barleymap database was used to find gene information within each breeder’s MQTL region (Cantalapiedra et al., 2015). Considering the international leadership in rice genomics research, a comparative genomics approach was employed to identify homologs that potentially influence rice quality traits within the breeder’s MQTL region of barley. Genes controlling quality-related traits in rice were searched at the China Rice Data Center (https://www.ricedata.cn/).

## Results

### Characteristics of QTL studies for quality traits in barley

**Figure 1:**
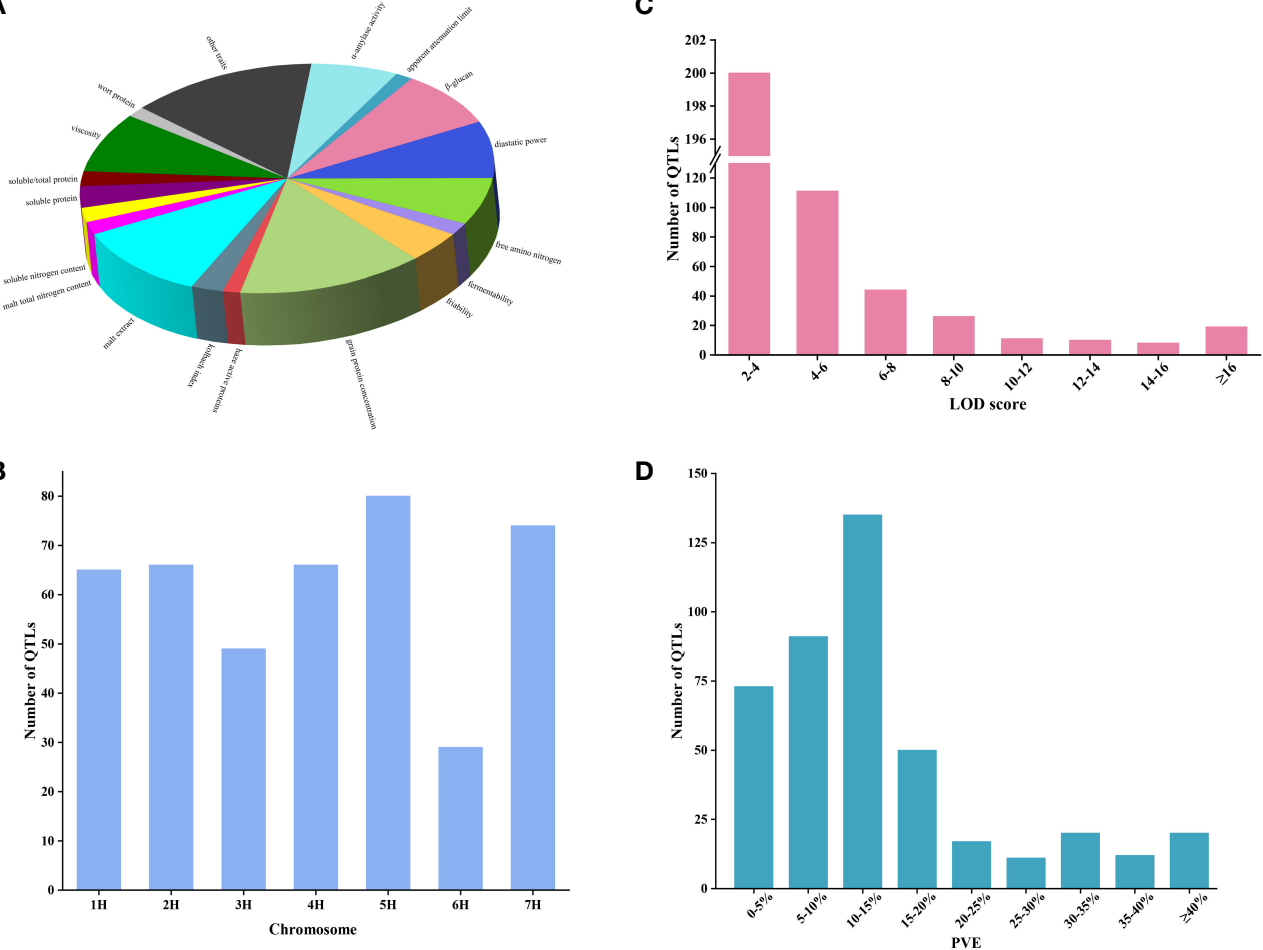
The QTL information for barley quality traits in previous QTL studies. **(A)** Percentage of QTL for different quality traits. **(B)** Distribution of QTL on seven chromosomes. **(C)** Frequency distribution of QTL with different LOD scores. **(D)** Frequency distribution of QTL for different PVE.

A comprehensive analysis was conducted on 35 QTL studies focusing on quality traits in barley from the year 2000 onwards. The details can be found in **Supplementary Table S1**. A total of 454 QTLs for quality traits in barley were collected from 39 mapping populations in 35 studies (**Supplementary Table S2**). These quality traits encompassed 42 different types, mainly focusing on malting quality traits, with a few additional traits such as flour pasting properties traits (**Supplementary Table S3**). The number of QTL varied for different barley quality traits. Higher numbers of QTL were identified for grain protein concentration (GPC), malt extract (ME), β-glucan (BG) diastatic power (DP), and viscosity (VIS), accounting for 14.2%, 11.4%, 8.6%, 8.2%, and 8.2% of the total QTL number, respectively. The remaining quality traits accounted for a lower percentage (**Figure 1A**). The distribution of these initial QTLs was uneven across all chromosomes. Chromosome 6H had the lowest number of QTLs, accounting for 6.8% (31/454), while the remaining chromosomes ranged from 52 to 83 QTLs (**Figure 1B**). Individual QTL exhibited LOD scores ranging from 2 to 64.55, with the majority falling within the range of 2 to 4 (**Figure 1C**). The PVE of single QTL ranged from 1.1% to 77.5%, with the majority of QTL ranging from 0-5% (17%), 5-10% (21.2%), and 10-15% (31.5%) (**Figure 1D**).

### Construction of a consensus genetic map in barley

A reference genetic map for barley was constructed by integrating six widely available barley genetic maps using the R package LPmerge. Subsequently, ten individual genetic maps were mapped to the reference genetic map by BioMercator v4.2 software, resulting in a high-density consensus genetic map for barley. This consensus map consisted of 28,382 markers, with a total length of 1146.53 cM and an average chromosome length of 163.79 cM (**Supplementary Table S4**; **Supplementary Table S7**; **Supplementary Tabl** **e S8**). The distribution of markers on each chromosome was not uniform, with chromosome 4H having the lowest number of markers (2970) and 2H having the highest number of markers (5115). The marker density varied across chromosome, with chromosome 2H having the highest marker density (29.57/cM) and chromosome 4H having the lowest density (20.07/cM). On average, the genetic distance between markers was found to be 0.04 cM (**Supplementary Table S8**; **Supplementary Figure 1**).

### Meta-QTL identification for quality traits

**Figure 2:**
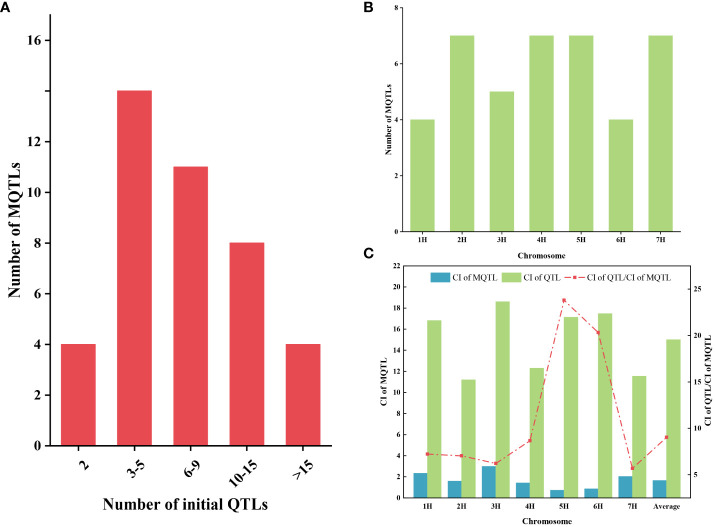
The basic information of MQTL was obtained by QTL meta-analysis. **(A)** Distribution of the number of MQTL containing different initial QTL numbers. **(B)** Distribution of MQTL on seven chromosomes. **(C)** Comparison of confidence intervals between initial QTL (green bars) and MQTL (blue bars). The red dotted line represents the narrowed fold of the QTL confidence intervals.

A total of 349 QTLs were projected onto the consensus map after excluding initial QTL with LOD value less than three and PVE missing from the 454 initial QTLs collected from 35 previous studies. Among these, 344 QTLs were integrated into 41 MQTLs through meta-analysis, while five QTLs did not overlap with any of the MQTLs mentioned above (**Supplementary Table S9**). Each MQTL contained a varying number of initial QTL, ranging from 2 to 38. Notably, 37 (90.2%) of the MQTLs consisted of three or more QTLs, with 12 MQTLs contained at least ten initial QTLs (**Figure 2A**). The distribution of these MQTLs was uneven across chromosomes, with the number ranging from four (1H and 6H) to seven (2H, 4H, 5H, and 7H) (**Figure 2B**). The average CI of MQTL on chromosomes ranged from 0. 72 (5H) to 2.99 (3H), whereas the CI of the initial QTL ranged from 11.19 (2H) to 18.6 (3H). The average CI of MQTL shrank by 9.04-fold compared with that of the initial QTL. The degree of reduction in the average CI of the MQTL varied across different chromosomes, with the smallest decrease (5.68-fold) observed on chromosome 7H and the largest decrease (23.79-fold) on chromosome 5H (**Figure 2C**).

### Validation of MQTL using previous GWAS studies

**Figure 3:**
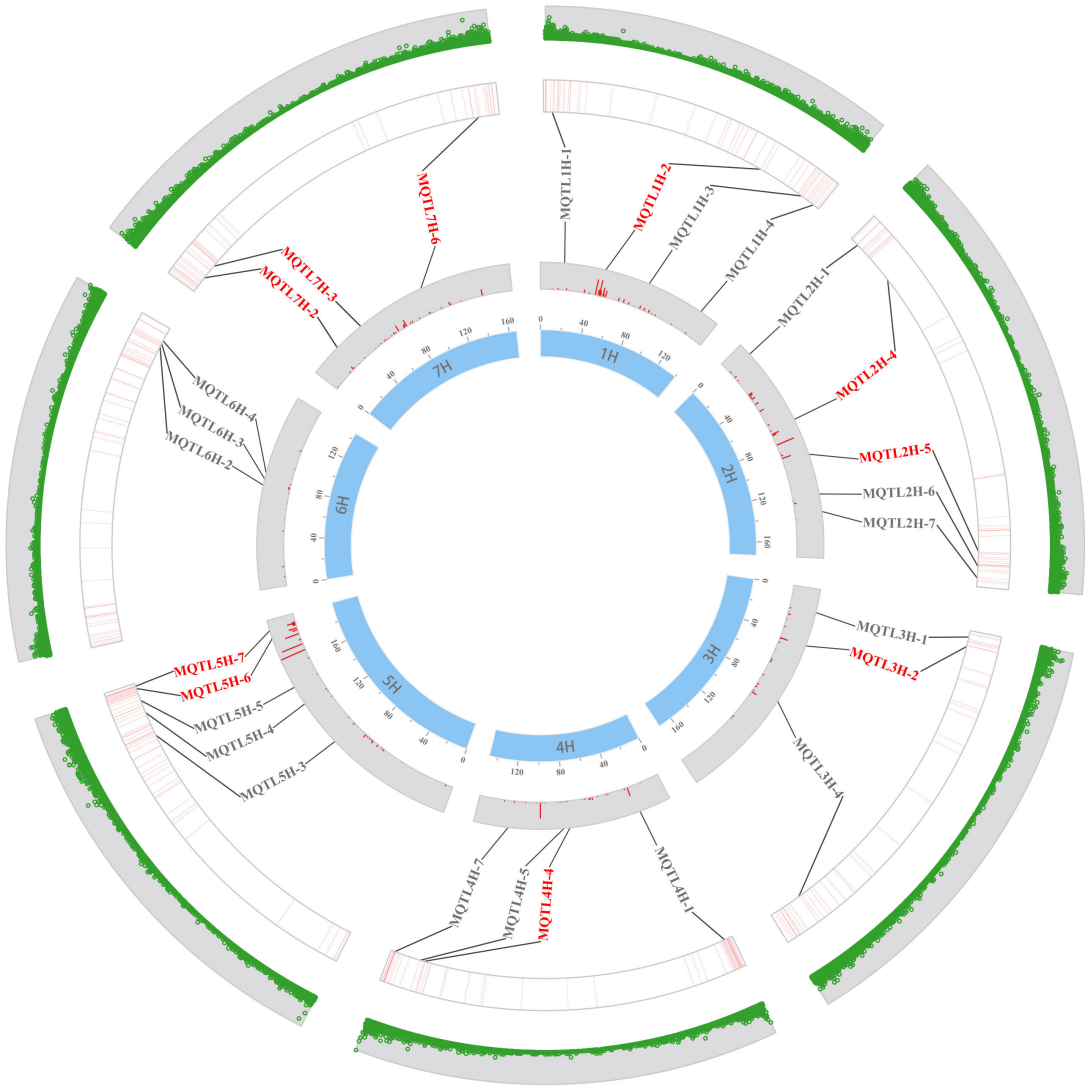
Distribution of MQTL on seven chromosomes verified by GWAS-MTAs in recent years. The inner to outer circles indicate the genetic map, the PVE of the initial QTL, the MTA’s position on the physical map, and the distribution of high-confidence genes, respectively. The red MQTLs indicate the breeder’s MQTLs.

In this study, 36 MQTLs mapped to the barley reference genome, with 27 of them localized in physical regions less than 20 Mb. To verify the accuracy of these MQTLs, we compared the physical locations of these MQTLs with the GWAS-MTAs for barley quality traits in recent years. Out of the 27 MQTLs, 25 were found to co-localized with MTAs in at least one GWAS study, and 12 MQTLs were validated in at least two studies. Notably, MQTL3H-1, MQTL4H-1, MQTL4H- 7, MQTL5H-3 and MQTL7H-6 were detected no less than three times in nine GWAS studies (**Supplementary Table S10**). In addition, several MQTLs, such as MQTL5H-6, MQTL5H-7, MQTL6H-2, MQTL6H-3, and MQTL6H-4 were clustered in the barley reference genome (**Figure 3**). Base on the criteria developed by Löffler et al. (2009), ten breeder’s MQTLs were screened from the 25 MQTLs validated by GWAS (**Supplementary Table S9**). These breeder’s MQTLs were predominantly located in the sub-telomeric regions of chromosomes and exhibited better co-linearity between the physical and genetic maps. Most of the breeder’s MQTLs affected multiple malt quality traits simultaneously. For example, MQTL2H-5, MQTL3H-2, and MQTL5H-6 were associated with different malt quality traits, while MQTL1H- 2 was related to both malt quality and flour pasting properties traits.

### Identification of orthologous MQTL in wheat

To identify OrMQTL in wheat, ten breeder’s MQTLs with physical intervals less than 20 Mb were selected. Among these MQTLs, two had no corresponding OrMQTL in wheat, while the remaining eight breeder’s MQTL had 45 orthologous wheat MQTLs (**Supplementary Table S11**). For example, MQTL1H-2 was isogenic to three wheat MQTLs (MQTL1A.5, MQTL1B.4, and MQTL1B.6), MQTL7H-3 was isogenic to four wheat MQTLs located on chromosomes 7B and 7D (MQTL7B.5, MQTL7B.6, MQTL7D.3, and MQTL7D.5), and MQTL7H-6 shared similarity with up to 25 wheat MQTLs. The number of conserved genes between barley and wheat MQTLs ranged from 4 (MQTL4A.1) to 313 (MQTL7B.3) (**Supplementary Table S11**).

### Candidate gene prediction within breeder’s MQTLs

Ten breeder’s MQTLs were screened for candidate gene (CGs) prediction based on the criteria for breeder’s MQTLs selection, combined with MQTL physical distances of less than 20 Mb (**Supplementary Table S7**). These breeder’s MQTLs were found to impact multiple barley quality traits simultaneously, suggesting the presence of significant candidate genes that regulate these traits within these regions. We searched for candidate genes within each breeder’s MQTL using the locate by position tool from Barleymap database (Cantalapiedra et al., 2015). Among the 800 gene models identified within the breeder’s MQTLs regions, MQTL7H- 6 had the highest number of 348 gene models, while other MQTLs ranged from 2 (MQTL2H-4) to 248 (MQTL3H-2) (**Supplementary Table S12**). Additionally, we identified 17 barley homologs of rice quality trait-related genes within the breeder’s MQTL regions by comparing homologous relationships with rice. These genes encode various products, mainly including aldehyde oxidase, protein kinase, cell-cycle protein, hexokinase, and shikimate kinase. They play roles in regulating traits such as anther dehiscence, pollen germination, anthocyanin biosynthesis, seed germination, and sucrose accumulation in rice (**Supplementary Table S13**).

## Discussion

### MQTL characterization for quality traits in barley

Barley, one of the important grain crops, possesses quality traits that impact food processing and beer brewing (Kochevenko et al., 2018). Recent years have witnessed numerous QTL mapping studies on quality traits, revealing the genetic mechanisms regulating barley quality (**Supplementary Table S1**). This study represents the first meta-analysis of QTLs for quality traits in barley, incorporating 454 QTLs collected from 35 independent studies, with more than 76% of the individual QTLs utilized for detecting MQTLs. Furthermore, this study constructed a consensus map containing a higher number and type of molecular markers than those developed in previous studies (Zhang et al., 2017; Khahani et al., 2019; Akbari et al., 2022). However, due to the limited number of AFLP and RFLP markers in the consensus map, there were still a few QTLs that could not be mapped to the consensus map.

Previous studies have accumulated a substantial number of QTLs for barley quality traits, but many of them exhibit low effects and wide confidence intervals, limiting their applicability for molecular marker-assisted selection. Moreover, the genetic location and effects of QTLs identified in different populations vary, hindering their utilization in genetic improvement. However, our study shows that the confidence intervals of MQTL were narrowed by 9.04-fold compared to the initial QTL (**Figure 2C**). The effectiveness of MQTLs in reducing confidence intervals was also confirmed in meta-analysis of quality traits in wheat and rice (Peng et al., 2021; Gudi et al., 2022). The reliability of MQTLs was significantly correlated with the number of initial QTLs included (Quraishi et al., 2017). In this study, we identified a total of 41 MQTLs, with over 90.2% (37/41) of th em containing more than three initial QTLs. Among these, 21 MQTLs comprised no less than seven QTLs (**Supplementary Table S9**).

Regarding the collected quality trait categories, the most commonly identified MQTLs were those related to grain protein concentration (GPC), malt extract (ME), β-glucan (BG), and viscosity (VIS). These traits are regulated by multiple genes in diverse conditions (Igartua et al., 2002; Fang et al., 2019).

The distribution of QTL is mainly influenced by gene density, polymorphism rate of functional loci, and recombination rate (Martinez et al., 2016). The sub-telomeric region of chromosomes in the barley genome contained the highest number of genes and exhibits a higher recombination rate (Mascher et al., 2017; Mascher et al., 2021). Coincidentally, most of the MQTL in this study were found in the sub-telomeric region of chromosomes (**Supplementary Table S9**). Similar findings have been reported in previous studies on MQTL for yield-related traits in barley (Khahani et al., 2019).

### Validation of MQTLs with GWAS

In addition to the traditional QTL linkage analysis, GWAS plays a significant role in identifying QTL for quantitative traits. Multiple studies on QTL meta-analysis have been conducted to validate MQTL results using GWAS data (Selamat and Nadarajah, 2021; Yang et al., 2021; Karnatam et al., 2023). In this study, 25 MQTLs were validated through GWAS studies on barley quality traits in recent years. Among these, 12 MQTLs co-localized with MTAs in no fewer than two GWAS studies, while five MQTLs (MQTL3H-1, MQTL4H-1, MQTL4H-7, MQTL5H-3 and MQTL7H-6) were validated in at least three GWAS studies (**Supplementary Table S10**), suggesting that these MQTLs may be important genomic regions involved in the regulation of barley quality traits. In addition, ten breeder’s MQTLs were screened out of the GWAS-validated MQTLs. Most of these breeder’s MQTLs were associated with multiple quality traits, such as MQTL1H-2, MQTL2H-4, MQTL3H-2, and MQTL5H-6, which affected no less than nine quality traits simultaneously. Identifying these breeder MQTLs provided useful information for accurately identifying candidate genes related to barley quality.

### OrMQTLs for wheat

The analysis of OrMQTLs has not been studied in barley, but in recent years, several studies have been reported for heat tolerance (Kumar et al., 2020), yield-related traits (Saini et al., 2022), grain zinc and iron contents (Shariatipour et al., 2021), and quality traits (Gudi et al., 2022) in wheat. In this study, we identified 45 conserved MQTLs in the breeder’s MQTL region that were associated with quality traits between barley and wheat (**Supplementary Table S11**). A few conserved genes in OrMQTL have been identified with certain quality traits in wheat. For example, barley MQTL3H-2 has a homolog *TaNAC019-3D* (*HORVU.MOREX.r3.3HG0231360*) in the syntenic region MQTL3D.3 in wheat, encoding a NAC domain-containing protein, which regulates gluten and starch accumulation and improves wheat grain quality (Gao et al., 2021). In addition, the gene *HORVU.MOREX.r3.7HG0740600* in the MQTL7H-6 region of barley has a homolog in the isogenic region of wheat (MQTL7B.3, MQTL7B.4, and MQTL7B.6) called *TaCol-B5*. This gene encodes a CONSTANS-like protein that affects the spikelet structure of wheat and improves wheat grain yield (Zhang et al., 2022). The analysis of OrMQTLs using gene homology between cereals revealed conserved regions between barley and wheat. These regions contain many uncharacterized or characterized genes that may be associated with quality traits in barley.

### Barley homologs of known rice genes within breeder’s MQTLs region

Barley has a significantly large genome compared to other grass crops, making it ideal for comparative genomics strategies in identifying candidate genes for important traits. Rice, as a well-studied model plant in the grass family, has advanced genomics research and gene cloning, making the analysis of homologous barley and rice genes valuable in identifying candidate genes for important traits in barley (Gaut, 2002). For example, the rice gene *OsRSR1*, a member of the AP2/EREBP family transcription factor, affects grain starch synthesis (Fu and Xue, 2010), and its barley ortholog *HvAP2-18* is involved in regulating grain starch synthesis (Ding et al., 2021). In addition, several important genes in barley demonstrated function similarly to their rice orthologs, such as *HvCKX6*, *HvGA20ox2*, *Ppd-H1*, and so on (Turner et al., 2005; Han et al., 2016; Xu et al., 2017).

In this study, 800 gene models were detected in the region of ten breeder’s MQTLs (**Supplementary Table S12**). From these models, a total of 17 barley homologs associated with rice quality traits were identified, suggesting their potential as candidate genes affecting barley quality (**Supplementary Table S13**). One such candidate gene is *HORVU.MOREX.r3.1HG0059550*, the barley ortholog of the rice gene *OsHXK10*, which was located in the MQTL1H- 2 region and encoded phosphotransferase. The gene *OsHXK10* encoding hexokinase was important in pollen germination and grain filling in rice (Xu et al., 2008). Thus, *HORVU.MOREX.r3.1HG0059550* is a reliable candidate gene for influencing quality traits in barley. Another example is *OsLOGL5*, a rice gene encoding uncharacterized protein PA4923, which was involved in seed development and grain filling processes (Wang et al., 2019). The barley ortholog *HORVU.MOREX.r3.5HG0536910*, located in the MQTL5H-6 region, encodes cytokinin riboside 5’-monophosphate phosphoribohydrolase, which may be a candidate gene affecting quality traits in barley. The rice gene *OsAO3* encodes aldehyde oxidase, which is expressed in relatively high amounts in germinating seeds, roots, and leaves, and regulates plant growth and seed yield by participating in ABA biosynthesis (Shi et al., 2021). The barley homeolog *HORVU.MOREX.r3.7HG0743390*, *HORVU.MOREX.r3.7HG0743420* and *HORVU.MOREX.r3.7HG0743430*, located in the MQTL7H-6 region, encode aldehyde oxidase and may be candidate genes involved in the regulation of quality traits in barley. The remaining 10 rice genes mainly encode mangiferyl kinase, protein kinase, glycosyl hydrolase, NAC transcription factor, etc., which are involved in the regulation of plant height, grain size, pollen germination, seed development, sucrose accumulation, and other traits. The 12 barley orthologs of these rice genes may also be candidate genes for quality traits in barley (**Supplementary Table S13**).

## Conclusions

In conclusion, we mapped previous QTLs for barley quality traits onto a high-density consensus map. Through QTL meta-analysis, we identified MQTLs, breeder’s MQTLs, and candidate genes, which deepens the understanding of the genetic mechanism underlying barley quality traits. In this study, we identified a total of 41 MQTLs, and compared with the initial QTL, the average CI of these MQTLs was reduced by 9.04-fold. Furthermore, 25 MQTLs were validated through GWAS-MTA. From the validated MQTLs, ten breeder’s MQTLs that can be utilized for molecular marker-assisted selection of barley quality traits were screened out. The identification of OrMQTLs in this study could be useful in understanding the conserved regions of between barley and wheat quality traits. Moreover, 17 barley homologs affecting quality traits were identified within the breeder’s MQTLs region based on comparing the homology of conserved genes in rice and barley. The se findings provide valuable information for the genetic improvement of quality traits and identification of candidate genes in barley.

## Data availability statement

The original contributions presented in the study are included in the article/**Supplementary Material**. Further inquiries can be directed to the corresponding authors.

## Author contributions

BD: Writing – original draft, Writing – review & editing, Formal analysis, Software. JDW: Data curation, Writing – original draft. MW: Data curation, Writing – original draft. JW: Software, Writing – original draft. CS: Data curation, Writing – original draft. XZ: Data curation, Writing – original draft. XR: Data curation, Writing – original draft. QW: Formal analysis, Writing – original draft, Writing – review & editing, Supervision.

## References

[B1] Akbari M. Sabouri H. Sajadi S. J. Yarahmadi S. Ahangar L. Abedi A.. (2022). Mega meta-QTLs: a strategy for the production of golden barley (*Hordeum vulgare* L.) tolerant to abiotic stresses. Genes. 13, 2087. doi: 10.3390/genes13112087 36360327 PMC9690463

[B2] Baik B. K. Ullrich S. E. (2008). Barley for food: Characteristics, improvement, and renewed interest. J. Cereal Sci. 48, 233–242. doi: 10.1016/j.jcs.2008.02.002

[B3] Bayer M. M. Rapazote-Flores P. Ganal M. Hedley P. E. Macaulay M. Plieske J.. (2017). Development and evaluation of a barley 50k iselect SNP array. Front. Plant Sci. 8. doi: 10.3389/fpls.2017.01792 PMC5651081 29089957

[B4] Brennan C. S. Harris N. Smith D. Shewry P. R. (1996). Structural differences in the mature endosperms of good and poor malting barley cultivars. J. Cereal Sci. 24, 171–177. doi: 10.1006/jcrs.1996.0050

[B5] Burger W. C. Laberge D. E. (1985). “Malting and brewing quality,” in Barley. Agron Mono, vol. 26 . Ed. Rasmussen D. C. (Madison, Wisconsin: ASA, CSSA, and SSSA), 367–401.

[B6] Cantalapiedra C. P. Boudiar R. Casas A. M. Igartua E. Contreras-Moreira B. (2015). BARLEYMAP: physical and genetic mapping of nucleotide sequences and annotation of surrounding loci in barley. Mol. Breeding. 35, 13. doi: 10.1007/s11032-015-0253-1

[B7] Close T. J. Bhat P. R. Lonardi S. Wu Y. Rostoks N. Ramsay L.. (2009). Development and implementation of high-throughput SNP genotyping in barley. BMC Genomics 10, 582. doi: 10.1186/1471-2164-10-582 19961604 PMC2797026

[B8] Coles G. D. Jamieson P. D. Haslemore R. M. (1991). Effect of moisture stress on malting quality in triumph barley. J. Cereal Sci. 14, 161–177. doi: 10.1016/S0733-5210

[B9] Daba S. Horsley R. Schwarz P. Chao S. Capettini F. Mohammadi M. (2018). Association and genome analyses to propose putative candidate genes for malt quality traits. J. Sci. Food Agr. 99, 2775–2785. doi: 10.1002/jsfa.9485 30430569

[B10] Darvasi A. Soller M. (1997). A simple method to calculate resolving power and confidence interval of QTL map location. Behav. Genet. 27, 125–132. doi: 10.1023/A:1025685324830 9145551

[B11] Ding J. J. Karim H. Li Y. L. Harwood W. Guzmán C. Lin N.. (2021). Re-examination of the APETALA2/Ethylene-responsive factor gene family in barley (*Hordeum vulgare* L.) indicates a role in the regulation of starch synthesis. Front. Plant Sci. 12. doi: 10.3389/fpls.2021.791584 PMC8672199 34925430

[B12] Du B. B. Wu J. Islam M. S. Sun C. Y. Lu B. W. Wei P. P.. (2022). Genome-wide meta-analysis of QTL for morphological related traits of flag leaf in bread wheat. PloS One 17, e0276602. doi: 10.1371/journal.pone.0276602 36279291 PMC9591062

[B13] Egger M. Smith G. D. Phillips A. N. (1997). Meta-analysis: principles and procedures. BMJ. 315, 1533–1537. doi: 10.1136/bmj.315.7121.1533 9432252 PMC2127925

[B14] Endelman J. B. Plomion C. (2014). LPmerge: an R package for merging genetic maps by linear programming. Bioinformatics. 30, 1623–1624. doi: 10.1093/bioinformatics/btu091 24532720

[B15] Fang Y. X. Zhang X. Q. Xue D. W. (2019). Genetic analysis and molecular breeding applications of malting quality QTLs in barley. Front. Genet. 10. doi: 10.3389/fgene.2019.00352 PMC6491634 31068969

[B16] Fox G. P. Panozzo J. F. Li C. D. Lance R. C. M. Inkerman P. A. Henry R. J. (2003). Molecular basis of barley quality. Aust. J. Agric. Res. 54, 1081–1101. doi: 10.1071/AR02237

[B17] Fu F. F. Xue H. W. (2010). Coexpression analysis identifies Rice Starch Regulator1, a rice AP2/EREBP family transcription factor, as a novel rice starch biosynthesis regulator. Plant Physiol. 154, 927–938. doi: 10.1104/pp.110.159517 20713616 PMC2949045

[B18] Gao W. Clancy J. A. Han F. Jones B. L. Budde A. Wesenberg D. M.. (2004). Fine mapping of a malting-quality QTL complex near the chromosome 4H S telomere in barley. Theor. Appl. Genet. 109, 750–760. doi: 10.1007/s00122-004-1688-7 15164174

[B19] Gao Y. J. An K. X. Guo W. W. Chen Y. M. Zhang R. J. Zhang X.. (2021). The endosperm-specific transcription factor *TaNAC019* regulates glutenin and starch accumulation and its elite allele improves wheat grain quality. Plant Cell. 33, 603–622. doi: 10.1093/plcell/koaa040 33955492 PMC8136912

[B20] Gaut B. S. (2002). Evolutionary dynamics of grass genomes. New Phytol. 154, 15–28. doi: 10.1046/j.1469-8137.2002.00352.x

[B21] Geng L. Li M. D. Xie S. G. Wu D. Z. Ye L. Z. Zhang G. P. (2021). Identification of genetic loci and candidate genes related to β-glucan content in barley grain by genome-wide association study in International Barley Core Selected Collection. Mol. Breeding. 41, 6. doi: 10.1007/s11032-020-01199-5 PMC10236047 37309529

[B22] Genievskaya Y. Almerekova S. Abugalieva A. Abugalieva S. (2021). Genome-wide association study of grain quality traits in spring barley collection grown in Kyzylorda region. Exp. Biol. 87, 36–47. doi: 10.26577/eb.2021.v87.i2.04

[B23] Genievskaya Y. Almerekova S. Abugalieva S. Abugalieva A. Sato K. Turuspekov Y. (2023). Identification of SNPs associated with grain quality traits in spring barley collection grown in southeastern Kazakhstan. Agronomy. 13, 1560. doi: 10.3390/agronomy13061560

[B24] Genievskaya Y. Almerekova S. Abugalieva S. Chudinov V. Blake T. Abugalieva A.. (2022). Identification of SNP markers associated with grain quality traits in a barley collection (*Hordeum vulgare* L.) harvested in Kazakhstan. Agronomy. 12, 2431. doi: 10.3390/agronomy12102431

[B25] Goffinet B. Gerber S. (2000). Quantitative trait loci: A meta-analysis. Genetics. 155, 463–473. doi: 10.1093/genetics/155.1.463 10790417 PMC1461053

[B26] Gudi S. Saini D. K. Singh G. Halladakeri P. Kumar P. Shamshad M.. (2022). Unravelling consensus genomic regions associated with quality traits in wheat using meta-analysis of quantitative trait loci. Planta. 255, 115. doi: 10.1007/s00425-022-03904-4 35508739

[B27] Guo B. Sleper D. A. Lu P. Shannon J. G. Nguyen H. T. Arelli P. R. (2006). QTLs associated with resistance to soybean cyst nematode in soybean: Meta-analysis of QTL locations. Crop Sci. 46, 595–602. doi: 10.2135/cropsci2005.04-0036-2

[B28] Halladakeri P. Gudi S. Akhtar S. Singh G. Saini D. K. Hilli H. J.. (2023). Meta-analysis of the quantitative trait loci associated with agronomic traits, fertility restoration, disease resistance, and seed quality traits in pigeonpea (*Cajanus cajan* L.). Plant Genome. 16, e20342. doi: 10.1002/tpg2.20342 37328945 PMC12807056

[B29] Han M. Wong J. L. Su T. Beatty P. H. Good A. G. (2016). Identification of nitrogen use efficiency genes in barley: searching for QTLs controlling complex physiological traits. Front. Plant Sci. 7. doi: 10.3389/fpls.2016.01587 PMC5073129 27818673

[B30] Hayter A. Riggs T. (1973). Environmental and varietal differences in diastatic power and four associated characteristics of spring barley. J. Agric. Sci. 80, 297–302. doi: 10.1017/S0021859600057762

[B31] Huang Y. D. Yin L. Sallam A. H. Heinen S. Li L. Beaubien K.. (2021). Genetic dissection of a pericentromeric region of barley chromosome 6H associated with Fusarium head blight resistance, grain protein content and agronomic traits. Theor. Appl. Genet. 134, 3963–3981. doi: 10.1007/s00122-021-03941-9 34455452

[B32] Igartua E. Hayes P. M. Thomas W. T. B. Meyer R. Mather D. E. (2002). Genetic control of quantitative grain and malt quality traits in barley. J. Crop Prod. 5, 131–164. doi: 10.1300/J144v05n01_06

[B33] Jensen J. Turner H. Lachowiec J. Lutgen G. Yin X. S. Sherman J. (2023). Genetic dissection of endosperm hydration in malting barley (*Hordeum vulgare*). Plant Breeding. 142, 639–649. doi: 10.1111/pbr.13138

[B34] Karnatam K. S. Chhabra G. Saini D. K. Singh R. Kaur G. Praba U. P.. (2023). Genome-wide meta-analysis of QTLs associated with root traits and implications for maize breeding. Int. J. Mol. Sci. 24, 6135. doi: 10.3390/ijms24076135 37047112 PMC10093813

[B35] Khahani B. Tavakol E. Shariati J. V. (2019). Genome-wide meta-analysis on yield and yield-related QTLs in barley (*Hordeum vulgare* L.). Mol. Breeding. 39, 565. doi: 10.1007/s11032-019-0962-y

[B36] Khahani B. Tavakol E. Shariati V. Fornara F. (2020). Genome wide screening and comparative genome analysis for Meta-QTLs, ortho-MQTLs and candidate genes controlling yield and yield-related traits in rice. BMC Genomics 21, 294. doi: 10.1186/s12864-020-6702-1 32272882 PMC7146888

[B37] Kochevenko A. Jiang Y. Seiler C. Surdonja K. Kollers S. Reif J. C.. (2018). Identification of QTL hot spots for malting quality in two elite breeding lines with distinct tolerance to abiotic stress. BMC Plant Biol. 18, 106. doi: 10.1186/s12870-018-1323-4 29866039 PMC5987402

[B38] Kumar A. Kaur S. Rakshit S. Choudhary M. Das A. K. Kumar R. R. (2021). Meta-analysis of QTLs associated with popping traits in maize *(Zea mays* L.). PloS One 16, e0256389. doi: 10.1371/journal.pone.0256389 34411180 PMC8376040

[B39] Kumar A. Saripalli G. Jan I. Kumar K. Sharma P. K. Balyan H. S.. (2020). Meta-QTL analysis and identification of candidate genes for drought tolerance in bread wheat (*Triticum aestivum* L.). Physiol. Mol. Biol. Pla. 26, 1713–1725. doi: 10.1007/s12298-020-00847-6 PMC7415061 32801498

[B40] Löffler M. Schön C. C. Miedaner T. (2009). Revealing the genetic architecture of FHB resistance in hexaploid wheat (*Triticum aestivum* L.) by QTL meta-analysis. Mol. Breeding. 23, 473–488. doi: 10.1007/s11032-008-9250-y

[B41] Looseley M. E. Ramsay L. Bull H. Swanston J. S. Shaw P. D. Macaulay M.. (2020). Association mapping of malting quality traits in UK spring and winter barley cultivar collections. Theor. Appl. Genet. 133, 2567–2582. doi: 10.1007/s00122-020-03618-9 32506274 PMC7419451

[B42] Mansuri R. M. Shobbar Z. S. Jelodar N. B. Ghaffari M. Mohammadi S. M. Daryani P. (2020). Salt tolerance involved candidate genes in rice: an integrative meta-analysis approach. BMC Plant Biol. 20, 452. doi: 10.1186/s12870-020-02679-8 33004003 PMC7528482

[B43] Marcel T. C. Varshney R. K. Barbieri M. Jafary H. De Kock M. J. D. Graner A.. (2006). A high-density consensus map of barley to compare the distribution of QTLs for partial resistance to Puccinia hordei and of defence gene homologues. Theor. Appl. Genet. 114, 487–500. doi: 10.1007/s00122-006-0448-2 17115126

[B44] Marquez-Cedillo L. A. Hayes P. M. Jones B. L. Kleinhofs A. Legge W. G. Rossnagel B. G.. (2000). QTL analysis of malting quality in barley based on the doubled-haploid progeny of two elite North American varieties representing different germplasm groups. Theor. Appl. Genet. 101, 173–184. doi: 10.1007/s001220051466

[B45] Martinez A. K. Soriano J. M. Tuberosa R. Koumproglou R. Jahrmann T. Salvi S. (2016). Yield QTLome distribution correlates with gene density in maize. Plant Sci. 242, 300–309. doi: 10.1016/j.plantsci.2015.09.022 26566847

[B46] Mascher M. Gundlach H. Himmelbach A. Beier S. Twardziok S. O. Wicker T.. (2017). A chromosome conformation capture ordered sequence of the barley genome. Nature. 544, 427–433. doi: 10.1038/nature22043 28447635

[B47] Mascher M. Wicker T. Jenkins J. Plott C. Lux T. Koh C. S.. (2021). Long-read sequence assembly: a technical evaluation in barley. Plant Cell. 33, 1888–1906. doi: 10.1093/plcell/koab077 33710295 PMC8290290

[B48] Mohammadi M. Blake T. K. Budde A. D. Chao S. Hayes P. M. Horsley R. D.. (2015). A genome-wide association study of malting quality across eight U.S. barley breeding programs. Theor. Appl. Genet. 128, 705–721. doi: 10.1007/s00122-015-2465-5 25666272

[B49] Peng Y. L. Zhang X. Zou T. Fan W. Tang S. W. Li L. M.. (2021). Meta-analysis of Qtl associated with starch pasting viscosity in rice (*Oryza Sativa* L.). Bangladesh J. Bot. 50, 269–276. doi: 10.3329/bjb.v50i2.54082

[B50] Quraishi U. M. Pont C. Ain Q. Flores R. Burlot L. Alaux M.. (2017). Combined genomic and genetic data integration of major agronomical traits in bread wheat (*Triticum aestivum* L.). Front. Plant Sci. 8, 1843. doi: 10.3389/fpls.2017.01843 29184557 PMC5694560

[B51] Saini D. K. Srivastava P. Pal N. Gupta P. K. (2022). Meta-QTLs, ortho-meta-QTLs and candidate genes for grain yield and associated traits in wheat (*Triticum aestivum* L.). Theor. Appl. Genet. 135, 1049–1081. doi: 10.1007/s00122-021-04018-3 34985537

[B52] Sato K. Hisano H. Matsumoto S. Zhou T. S. Kihara M. (2018). Detection of QTLs controlling alpha-amylase activity in a diversity panel of 343 barley accessions. Mol. Breeding. 38, 14. doi: 10.1007/s11032-017-0773-y

[B53] Selamat N. Nadarajah K. K. (2021). Meta-analysis of quantitative traits loci (QTL) identified in drought response in rice (*Oryza sativa* L.). Plants. 10, 716. doi: 10.3390/plants10040716 33917162 PMC8067883

[B54] Shariatipour N. Heidari B. Richards C. M. (2021). Meta-analysis of QTLome for grain zinc and iron contents in wheat (*Triticum aestivum* L.). Euphytica. 217, 86. doi: 10.1007/s10681-021-02818-8 PMC8546302 34712248

[B55] Shi X. Tian Q. Deng P. Zhang W. Jing W. (2021). The rice aldehyde oxidase *OsAO3* gene regulates plant growth, grain yield, and drought tolerance by participating in ABA biosynthesis. Biochem. Bioph Res. Co. 548, 189–195. doi: 10.1016/j.bbrc.2021.02.047 33647795

[B56] Sosnowski O. Charcosset A. Joets J. (2012). BioMercator V3: an upgrade of genetic map compilation and quantitative trait loci meta-analysis algorithms. Bioinformatics. 28, 2082–2083. doi: 10.1093/bioinformatics/bts313 22661647 PMC3400960

[B57] Tanin M. J. Saini D. K. Sandhu K. S. Pal N. Gudi S. Chaudhary J.. (2022). Consensus genomic regions associated with multiple abiotic stress tolerance in wheat and implications for wheat breeding. Sci. Rep. 12, 13680. doi: 10.1038/s41598-022-18149-0 35953529 PMC9372038

[B58] Turner A. Beales J. Faure S. Dunford R. P. Laurie D. A. (2005). The pseudo-response regulator Ppd-H1 provides adaptation to photoperiod in barley. Science. 310, 1031–1034. doi: 10.1126/science.1117619 16284181

[B59] Varshney R. K. Marcel T. C. Ramsay L. Russell J. Roder M. S. Stein N.. (2007). A high density barley microsatellite consensus map with 775 SSR loci. Theor. Appl. Genet. 114, 1091–1103. doi: 10.1007/s00122-007-0503-7 17345060

[B60] Veyrieras J. B. Goffinet B. Charcosset A. (2007). MetaQTL: a package of new computational methods for the meta-analysis of QTL mapping experiments. BMC Bioinf. 8, 49. doi: 10.1186/1471-2105-8-49 PMC1808479 17288608

[B61] Von Korff M. Wang H. J. Léon J. Pillen K. (2008). AB-QTL analysis in spring barley: III. Identification of exotic alleles for the improvement of malting quality in spring barley (*H. vulgare* ssp. spontaneum). Mol. Breeding. 21, 81–93. doi: 10.1007/s11032-007-9110-1

[B62] Wang C. G. Wang G. K. Gao Y. Lu G. H. Habben J. E. Mao G. F.. (2019). A cytokinin-activation enzyme-like gene improves grain yield under various field conditions in rice. Plant Mol. Biol. 102, 373–388. doi: 10.1007/s11103-019-00952-5 31872309

[B63] Wang Y. J. Wang Y. L. Wang X. Deng D. X. (2020). Integrated meta-QTL and genome-wide association study analyses reveal candidate genes for maize yield. J. Plant Growth Regul. 39, 229–238. doi: 10.1007/s00344-019-09977-y

[B64] Wang X. L. Zhang X. L. Cai S. G. Ye L. Z. Zhou M. X. Chen Z. H.. (2015). Genetic diversity and QTL mapping of thermostability of limit dextrinase in barley. J. Agric. Food Chem. 63, 3778–3783. doi: 10.1021/acs.jafc.5b00190 25816850

[B65] Wenzl P. Li H. Carling J. Zhou M. Raman H. Paul E.. (2006). A high-density consensus map of barley linking DArT markers to SSR RFLP and STS loci and agricultural traits. BMC Genomics 7, 206. doi: 10.1186/1471-2164-7-206 16904008 PMC1564146

[B66] Xu Y. H. Jia Q. J. Zhou G. F. Zhang X. Q. Angessa T. Broughton S.. (2017). Characterization of the *sdw1* semi-dwarf gene in barley. BMC Plant Biol. 17, 11. doi: 10.1186/s12870-016-0964-4 28086794 PMC5237212

[B67] Xu F. Q. Li X. R. Ruan Y. L. (2008). RNAi-mediated suppression of hexokinase gene *OsHXK10* in rice leads to non-dehiscent anther and reduction of pollen germination. Plant Science. 175, 674–684. doi: 10.1016/j.plantsci.2008.07.002

[B68] Yang Y. Amo A. Wei D. Chai Y. Zheng J. Qiao P.. (2021). Large-scale integration of meta-QTL and genome-wide association study discovers the genomic regions and candidate genes for yield and yield-related traits in bread wheat. Theor. Appl. Genet. 134, 3083–3109. doi: 10.1007/s00122-021-03881-4 34142166

[B69] Zhang X. Y. Jia H. Y. Li T. Wu J. Z. Nagarajan R. Lei L.. (2022). *TaCol-B5* modifies spike architecture and enhances grain yield in wheat. Science. 376, 180–183. doi: 10.1126/science.abm0717 35389775

[B70] Zhang X. Shabala S. Koutoulis A. Shabala L. Zhou M. (2017). Meta-analysis of major QTL for abiotic stress tolerance in barley and implications for barley breeding. Planta. 245, 283–295. doi: 10.1007/s00425-016-2605-4 27730410

[B71] Zhou G. Zhang Q. Tan C. Zhang X. Q. Li C. (2015). Development of genome-wide InDel markers and their integration with SSR, DArT and SNP markers in single barley map. BMC Genomics 16, 804. doi: 10.1186/s12864-015-2027-x 26474969 PMC4609152

