# Supplementary material for: Detection of consensus genomic regions and candidate genes for quality traits in barley using QTL meta-analysis
Source: Front Plant Sci. 2024 Jan 11;14:1319889. doi: 10.3389/fpls.2023.1319889 (PMC10811794; doi:10.3389/fpls.2023.1319889)
Supplement: Supplementary file 1 [file DataSheet_1.docx]

# Supplementary Data 1

**R package LPmerge Script:**

library(LPmerge)

library(Rglpk)

setwd("C:/Users/Du/Desktop/Barley consensus map/consensus map")

map.names = c("B1", "C1", "M1", "V1", "W1", "Z1")

#############1

map.names = c("B1", "C1", "M1", "V1", "W1", "Z1")##1

Maps <- list()

i <- 1

for (i in 1:6) {

filename <- paste(map.names[i],".csv",sep="")

input <- read.csv(filename,header=T,as.is=T,check.names=F)

Maps[[i]] <- input[which(input$chr=="4H"), c(1,3)]

}

names(Maps) <- map.names

str(Maps)

print(link.map.lengths <- unlist(lapply(Maps,function(x){max(x$cM)})))

mean(link.map.lengths)

unweighted <- LPmerge(Maps,max.interval=1:4)

head(unweighted[[1]])

write.csv(unweighted[[1]], file = "CA_1.txt")

sink("out_CA_1.txt")

print(input$marker)

print(input$chr)

print(input$cM)

print("link.map.lengths results")

print("map.names results")

print("unweighted <- LPmerge(Maps,max.interval=1:4)")

print(unweighted)

sink()


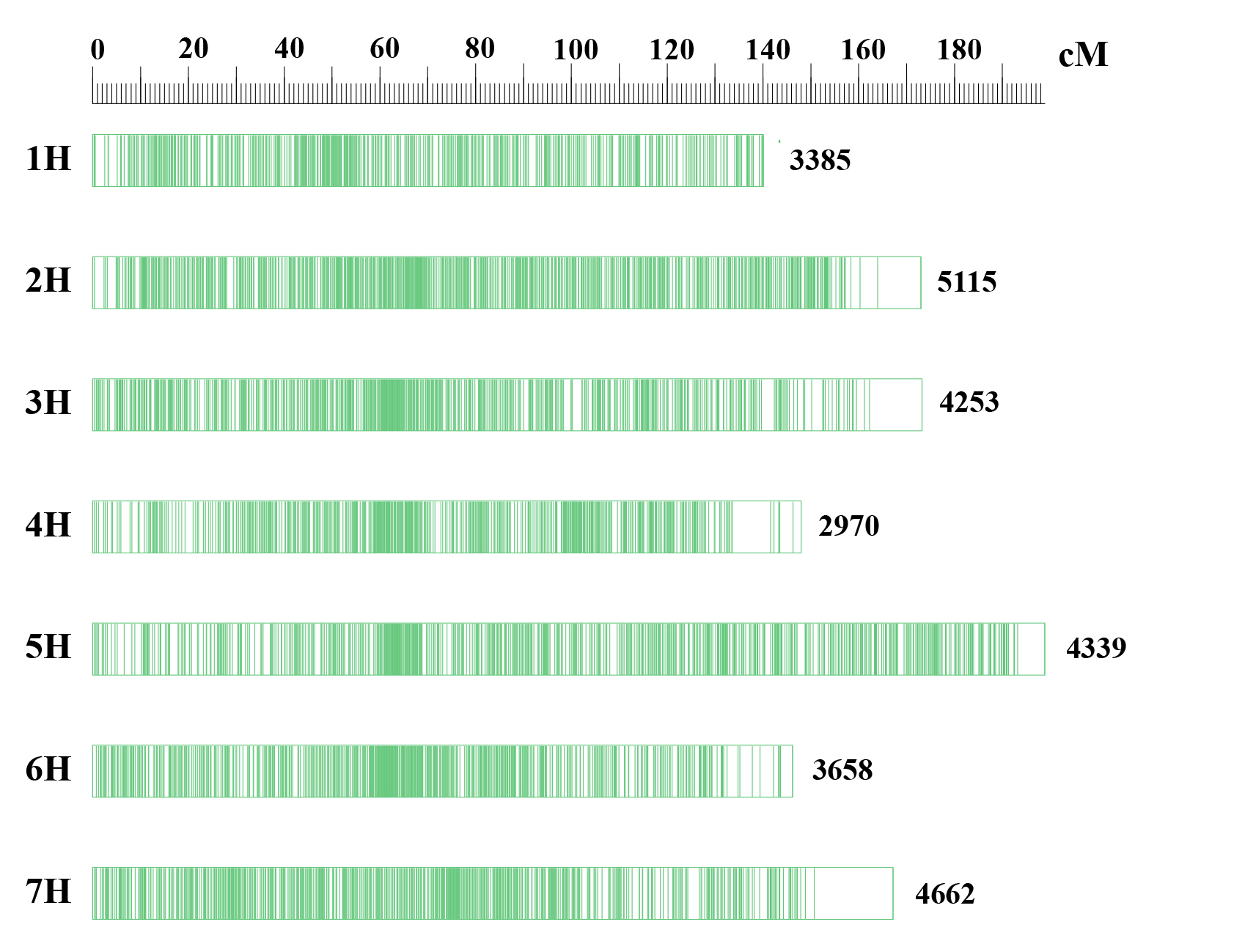


# Supplementary Figure 1. Distribution of molecular markers on the high-density consensus map in barley.
